# Phenotypic Craniofacial and Upper Spine Characteristics in Patients with Obstructive Sleep Apnoea

**DOI:** 10.3390/dj13030136

**Published:** 2025-03-20

**Authors:** Anne Marie Aavang Arvidson, Liselotte Sonnesen

**Affiliations:** Section for Orthodontics, Department of Odontology, Faculty of Health and Medical Sciences, University of Copenhagen, DK-2200 Copenhagen, Denmark; aacv@hvidovre.dk

**Keywords:** obstructive sleep apnoea, craniofacial morphology, head posture, upper spine morphological deviations

## Abstract

**Background/Objectives**: This study investigates differences in craniofacial morphology including skull thickness, sella turcica morphology, nasal bone length, and posterior cranial fossa dimensions, as well as differences in head posture and deviations in upper spine morphology, in adult OSA patients compared to healthy controls with neutral occlusion. **Methods**: 51 OSA patients (34 men, 17 women, mean age 51.9 ± 11.3 years) and 74 healthy controls (19 men, 55 women, mean age 38.7 years ± 14.0 years) with neutral occlusion were included. Craniofacial morphology and head posture were investigated using cephalometric measurements on lateral cephalograms and morphological deviations in sella turcica and upper spine were assessed through visual description of lateral cephalograms. **Results**: OSA patients had significantly more retrognathic maxilla (*p* = 0.02) and mandible (*p* = 0.032 and *p* = 0.009), significantly larger beta-angle (*p* = 0.006), and significantly smaller jaw angle (*p* = 0.045) compared to controls. OSA patients had significantly larger length (*p* = 0.003, *p* = 0.001, *p* = 0.044) and depth of the posterior cranial fossa (*p* < 0.001) compared to controls. OSA patients had a significantly more extended (*p* < 0.001) and forward-inclined head posture (*p* < 0.001) and morphological deviations in the upper spine occurred significantly more often in OSA patients compared to controls (*p* = 0.05). No significant differences in skull thickness, nasal bone length, and morphological deviations in the sella turcica (*p* = 0.235) were found between the groups. **Conclusions:** Significant deviations were found in craniofacial morphology, head posture, and morphological deviations in the upper spine. The results may prove valuable in the diagnostics of OSA patients and in considerations regarding etiology and the phenotypic differentiation of OSA patients.

## 1. Introduction

Obstructive Sleep Apnoea (OSA) is a common disease and the most frequent sleep-related breathing disorder [1,2,3]. Population studies have shown that the occurrence of mild OSA is up to 24.1% [4], while the occurrence of adults with moderate-to-severe OSA is 13% in males between 30–70 years and 6% in women between 30–70 years [5]. There is a tendency of increased occurrence of OSA in Western countries, presumably caused by increasing problems with being overweight [3,6].

OSA is caused by the complete or partial collapse of the upper airways during sleep, leading to changes in oxygen uptake as well as changes in the partial pressure of oxygen and carbon dioxide in the bloodstream [6]. The resulting decrease in oxygen pressure results in a disrupted sleep due to arousals, and patients with OSA experience pronounced daytime sleepiness during common activities such as driving, working, and during stationary activities [7]. The aetiology behind OSA is multifactorial, with age, gender, being overweight, and the genetically caused constriction of the upper airways as predisposing factors [2,8]. The occurrence of OSA is greater in patients with diabetes, heart diseases, Chronic Obstructive Pulmonary Disease, and different neurological disorders, as well as in patients with different craniofacial syndromes [1,2,9]. OSA is associated with increased systemic blood pressure and may lead to cardiovascular disease and diabetes [1,10,11]. Moreover, OSA is associated with increased general mortality risk [1].

The primary treatment options are Continuous Positive Airway Pressure (CPAP) and mandibular advancement device (MAD) as well as surgery of the upper airways [6,12,13].

Previous studies have shown that adult OSA patients phenotypically differ from healthy controls with regards to the upper spine. There has been found a significantly increased occurrence of morphological deviation of the upper spine in OSA patients [14,15], as well as an extended and forward-inclined head posture [15,16,17]. With regards to craniofacial morphology, the literature disagrees on whether there is a clear difference in the craniofacial morphology between OSA patients and controls [15]. Some studies have shown significantly more retrognathia of the maxilla and mandible, a more posterior-inclined mandible, and a larger jaw angle in OSA patients compared to controls [15,18,19,20], while other studies did not find any differences in the craniofacial morphology between OSA patients and controls [21]. Even though previous studies have investigated the craniofacial morphology in OSA patients, no previous studies have included skull thickness, sella turcica morphology, and the nasal bone length. Furthermore, it has been shown that craniofacial morphology and upper spine morphological deviations may play a role in the diagnostics and treatment of OSA patients [22].

Therefore, the present study investigates differences in the craniofacial morphology, including skull thickness, sella turcica morphology, nasal bone length, and posterior cranial fossa dimensions, as well as differences in head posture and deviations in upper spine morphology, in adult OSA patients compared to a healthy control group with neutral occlusion. The results from this study may thus contribute to finding new phenotypic characteristics in OSA patients and thereby contribute to the diagnostics and treatment of OSA patients.

## 2. Materials and Methods

### 2.1. Subjects

#### 2.1.1. OSA Patients

The data on the OSA patient group was systematically selected as part of standard material from 111 patients at the Dental Sleep Clinic, Section of Orthodontics, Department of Odontology at the University of Copenhagen. The OSA patients were diagnosed with OSA by ear–nose–throat medical doctors using polygraphy (Embletta, TK2, Natus Neurology Incorporated, Middleton, WI, USA), and subsequently referred for treatment at the Dental Sleep Clinic in the period of August 2018–February 2023.

The inclusion criteria were as follows:
  - Apnoea-Hypopnoea-Index (AHI) > 5;
  - Lateral cephalograms taken in natural head posture [18] at treatment start;
  - Height and weight measurements undertaken for measurement of body mass index (BMI).
The exclusion criteria were as follows:
  - AHI < 5, or lacking information about AHI;
  - Missing lateral cephalograms;
  - Missing measurements of height and weight.

**Figure 1:**
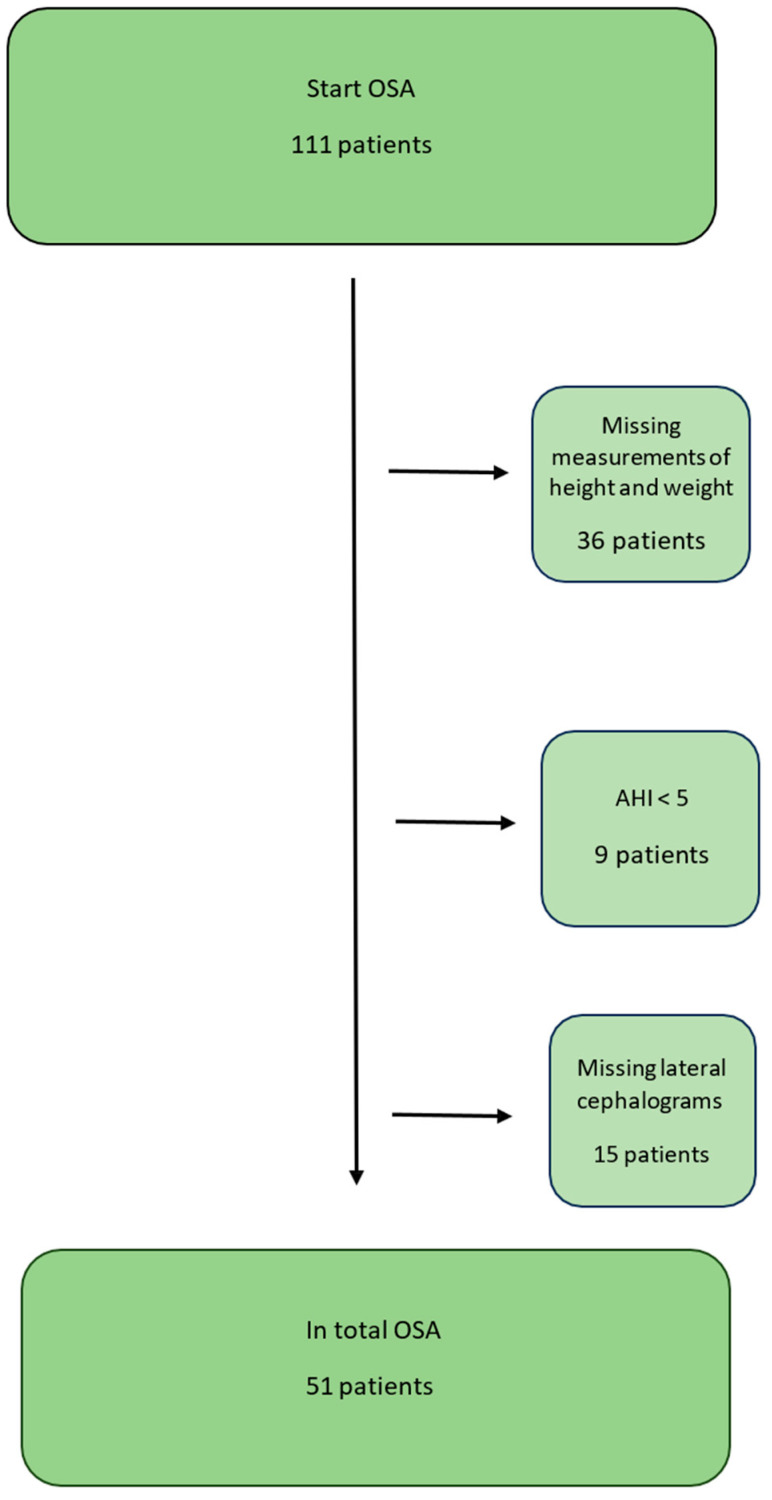
Flow chart for the OSA group.

The OSA group included 51 subjects, 17 women and 34 men, aged 26–73 years (mean age: 51.9 ± 11.3 years) (Figure 1). AHI was in an interval between 7 and 57 (mean AHI 21.8 ± 10.84) and BMI was between 18.4 and 39.0 (mean BMI 26.74 ± 4.85).

#### 2.1.2. Control Group

The data on the control group was gathered as part of two previous studies of 75 healthy subjects at the Section of Orthodontics, Department of Odontology at the University of Copenhagen, in the periods 2017–2018 and 2022–2023. All the control subjects were included in the present study except one due to missing lateral cephalograms.

The inclusion criteria were as follows:
  - Neutral occlusion [23] with no previous orthodontic treatment;
  - Lateral cephalograms taken in natural head posture [24];
  - Height and weight measurements undertaken for measurement of BMI.
The exclusion criteria were as follows:
  - Previous orthodontic treatment;
  - Known craniofacial syndromes and/or other general diseases;
  - Known sleep or respiratory conditions;
  - Missing information about height and weight.

The control group in the present study consisted of 74 subjects, 55 women and 19 men, aged 20–71 years (mean age: 38.7 years ± 14.0 years), and with a BMI between 18.2 and 44.2 (mean BMI 24.15 ± 3.98).

The power calculation performed prior to the present study was based on previous studies on upper spine morphological deviations. It was found that upper spine morphological deviations occurred in 32–46% in OSA patients [14] and in 3–14% in healthy subjects [25,26]. Under the assumption that 41% of the OSA patients and 5.5% of the healthy subjects have upper spine morphological deviations, at least 22 subjects in each group were required to have sufficient power (80%) to identify statistically significant differences at the 5% level of significance.

The material from the OSA group is standard material for diagnostics and treatment planning from patients at the Dental Sleep Clinic, Department of Odontology, University of Copenhagen, which does not require ethical approval. The OSA group has given oral and written acceptance of using their material for scientific investigations. The material from the control group had previously been approved by the Danish National Committee for Health Research Ethics (ref. no. H-17015290 and H-22008426; Approved on 2 July 2022). Moreover, the present study was approved by the Danish Data Protection Agency (data: J. no. 2599656/4242; Approved on 30 July 2017).

### 2.2. Methods

The present study was a cross-sectional study where upper spine morphological deviations, head posture, and craniofacial morphology, including skull thickness, length- and depth measurements in the posterior cranial fossa, nasal bone length, and sella turcica morphology were investigated on lateral cephalograms in OSA patients compared to controls.

All the subjects had their lateral cephalograms taken at the Cephalometric Laboratory, Department of Odontology, University of Copenhagen, by an experienced X-ray technician. The lateral cephalograms were taken using Promax (2D X-ray-unit-Planmeca OY, Helsinki, Finland, 2012) with a film-focus-distance of 501 mm, a magnification of 13%, and a resolution of 183. The lateral cephalograms were taken while the subjects were standing in natural head posture, as described by Solow and Tallgren [24].

All the lateral cephalograms were analysed in Tiops5 (version 4.5.1.0, Total Interactive Orthodontic Planning System, TIOPS, Denmark), by which the craniofacial morphology, skull thickness, posterior cranial fossa dimensions, nasal bone length, and head posture were measured. Morphological deviations in the upper spine and deviations in sella turcica morphology were examined by visual assessment on lateral cephalograms.

#### 2.2.1. Cephalometric Analysis of Lateral Cephalograms

**Figure 2:**
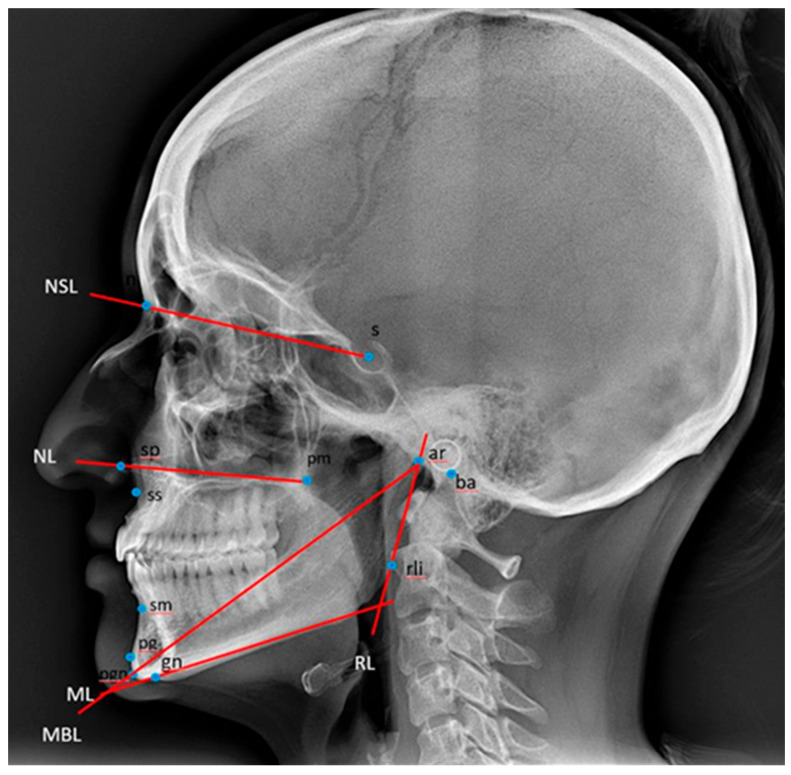
Reference points and lines used for the description of the craniofacial morphology [27]. ML: mandibular line, the tangent to the lower border of the mandible through gn. MBL: mandibular baseline, the line through pgn and ar. NL: nasal line, the line through sp and pm. NSL: nasion–sella line, the line through n and s. RL: ramus line, the tangent to the posterior border of the mandible. Ba: basion, the most postero-inferior point on the clivus. Ar: the point of intersection of the dorsal contours of the articular process of the mandible and the temporal bone. Gn: gnathion, the most inferior point on the mandibular symphysis. N: nasion, the most anterior point of the frontonasal suture. Pg: pogonion, the most anterior point of the mandibular symphysis. Pm: pterygomaxillare, the intersection between the nasal floor and the posterior contour of the maxilla. S: sella, the centre of the sella turcica, the upper limit of the sella turcica is defined as the line joining the tuberculum and the dorsum sellae. Sm: supramentale, the most posterior point on the anterior contour of the lower alveolar arch. Sp: spinal point, the apex of the anterior nasal spine. Ss: subspinale, the most posterior point on the anterior contour of the upper alveolar arch. Rli: ramus line inferior, the tangent point on the posterior border of the mandibular ramus to a line through ar. Pgn: Prognathion, the point on the mandibular symphysis with the maximum length from the condylion.

The cephalometric analysis was carried out according to Björk [27], and reference points and lines are described in Table 1 and illustrated in Figure 2.

**Figure 3:**
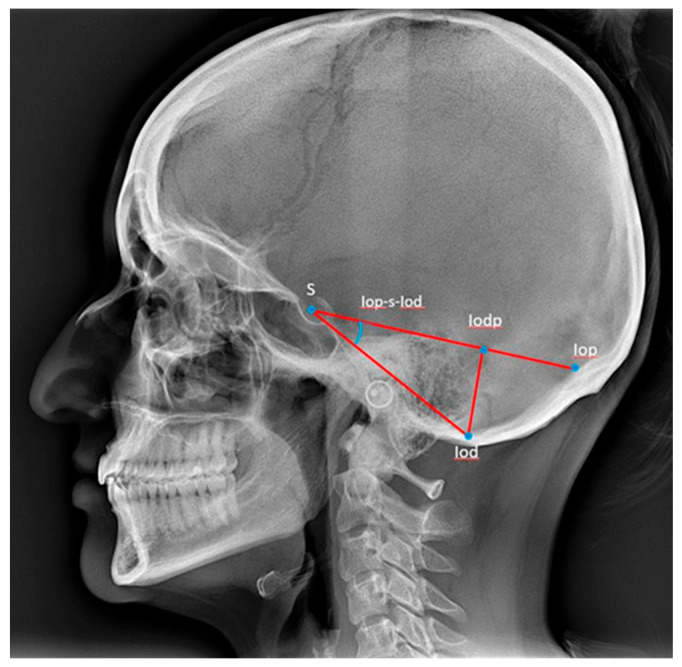
Reference points and lines used for the description of the posterior cranial fossa [28]. S: sella, the centre of the sella turcica. Iop: the internal occipital protuberance. Iod: the deepest point in the posterior cranial fossa. Iodp-Iop: the length from the internal occipital protuberance to the point Iodp. Iodp-Iod: the length from the point Iodp to the deepest point in the posterior cranial fossa. Iop-s-Iod: the angle between the Iop-S and the S-Iod lines. S-Iod: the length from sella to the deepest point in the posterior cranial fossa. S-Iop: the length from sella to the internal occipital protuberance.

The measurement of the posterior cranial fossa dimensions was carried out according to Caspersen et al. [28], and reference points and lines are described in Table 2 and illustrated in Figure 3 [28].

**Figure 4:**
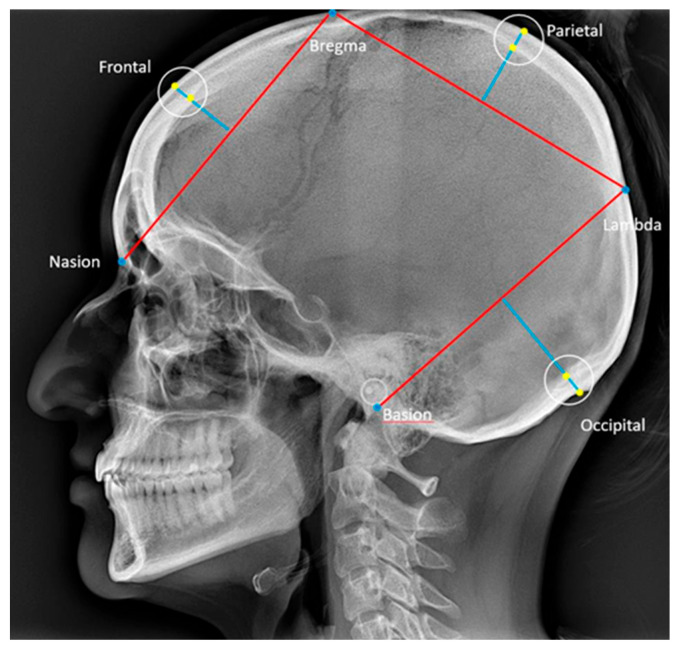
Reference points and lines used for the description of skull thickness in the frontal, parietal, and occipital bones [29]. Nasion: the most anterior point on the fronto-nasal suture. Basion: the most posterior-inferior point on the clivus. Bregma: the intersection between the sagittal and coronal sutures on the surface of the cranial vault. Lambda: the intersection between the lambdoid and sagittal sutures on the surfaces of the cranial vault. The thickness of the frontal, parietal, and occipital bones were defined according to Axelsson et al. [29] as the distances from the points where the perpendicular bisectors of the cords nasion–bregma, bregma–lambda, and lambda–basion intersected the inner and outer contours of the respective bones.

Skull thickness was measured frontally, parietally, and occipitally according to Axelsson [29], and the reference points and lines are described in Table 2 and illustrated in Figure 4 [29].

**Figure 5:**
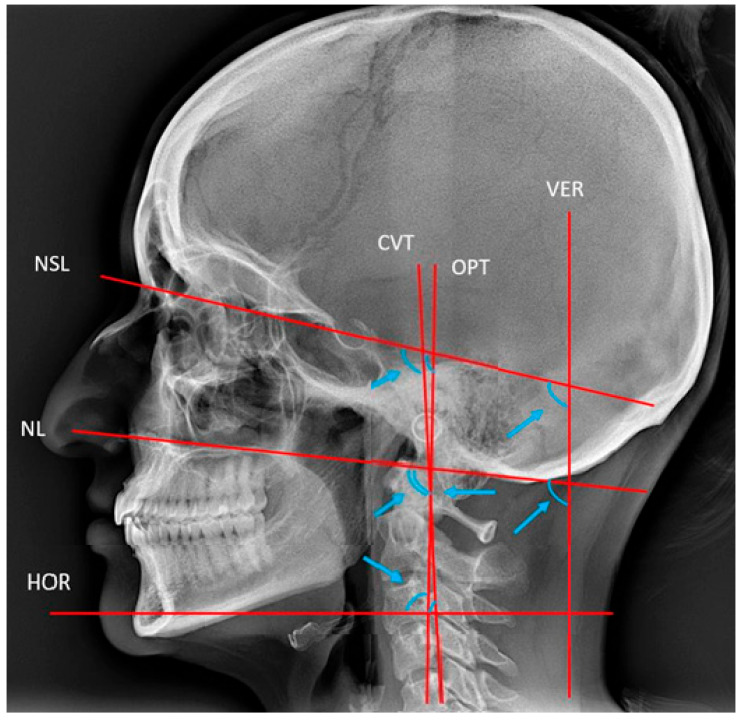
Reference lines and angles used for the description of head posture [19]. VER and HOR, true vertical and horizontal lines. CVT and OPT, cervical and odontoid process tangents. NL and NSL, nasal and nasion–sella lines. The arrow heads point to the angles NSL/VER, NSL/CVT, NSL/OPT, NL/CVT, NL/OPT, NL/VER, CVT/HOR, OPT/HOR, and the cervical curvature CVT/OPT.

Head posture was measured as described by Solow and Tallgren [24], and reference points and lines are described in Table 3 and illustrated in Figure 5 [24].

#### 2.2.2. Visual Analysis of Lateral Cephalograms

For each subject, a visual analysis of the lateral cephalogram has been conducted for the assessment of deviated sella turcica morphology, according to Axelsson et al. [30].

**Figure 6:**
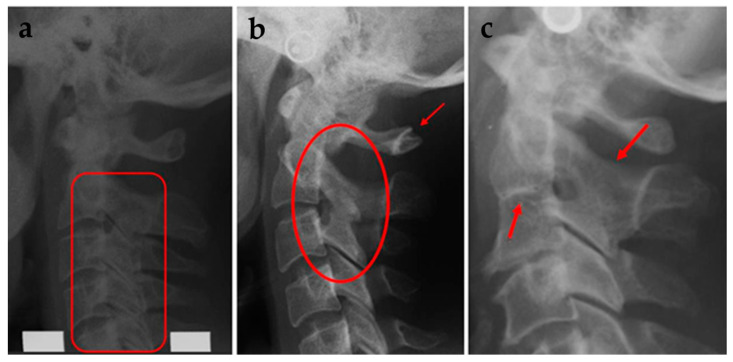
(**a**) Block fusion between C2-C3-C4-C5. (**b**) Fusion between C2-C3 and partial cleft of C1. (**c**) Fusion between corpus and arcus of C2-C3 [25].

For the assessment of morphological deviations in the upper spine, a visual analysis has been conducted of the first 5 cervical vertebrae. The deviations are classified in two groups, fusions (FUS) or posterior arch deficiencies (PAD), according to Sandham [25]. FUS was classified as fusion, block fusion, or occipitalisation [25] and PAD was classified as partial cleft or dehiscence of the cervical vertebrae (Figure 6). All the assessments were checked by LS, who is experienced in assessment of the upper spine.

### 2.3. Statistical Analysis

The statistical analysis was carried out using the statistics software IBM SPSS Statistics version 29.0.1.0 (SPSS Inc.; Chigaco, IL, USA), and the level of significance was set to 5% (*p* < 0.05). The normality of the distribution was assessed by quantile–quantile plots (Q-Q plots). All the continuous data for craniofacial morphology were normally distributed. Differences in age and BMI between the OSA group and the control group were examined through an independent *t*-test. Differences in gender between the groups were examined through a Chi-square test. The differences in craniofacial morphology, cranial fossa dimensions, skull thickness, nasal bone length, and head posture between the OSA group and the control group were analysed using general linear regression models adjusted for age, gender, and BMI. Differences in the occurrence of the categorical variables for upper spine morphological deviations and sella turcica morphological deviations between the groups were assessed by Fisher’s Exact test and adjusted age, gender, and BMI by logistic regression. Differences within the OSA patients grouped according to AHI severeness (mild, moderate, or severe) on both continuous and categorical variables were tested using a general linear model and logistic regression, adjusted for age, gender, and BMI. No significant differences were found between OSA patients with mild, moderate, or severe OSA. Therefore, the OSA group constitutes one single group before comparison with the control group.

### 2.4. Reliability

To determine the intra-examiner reliability, 25 lateral cephalograms were chosen randomly and analysed twice within 3 weeks. For the assessment of systematic error, a paired sampled *t*-test was performed. No systematic error was found. The method error for the craniofacial morphology including skull thickness and posterior cranial fossa dimensions ranged between 0.4° and 2.4°, and for the head posture between 0.7° and 2.2° assessed by Dahlberg’s formula [31]. Houston’s reliability coefficient [32] for the craniofacial morphology including skull thickness and posterior cranial fossa dimensions ranged between 0.89° and 0.99°, and for the head posture between 0.97° and 0.99°. The method error for the sella turcica morphology was 0.78 assessed by kappa [33]. Upper spine morphology was evaluated together with an experienced examiner (LS) and the reliability of the upper spine morphological deviations had previously been shown as excellent (k = 0.82) [14].

## 3. Results

The OSA patients were significantly older (*p* < 0.001) and had a BMI (*p* = 0.001) significantly larger compared to the controls. Significantly more men were included in the OSA group compared to the control group (*p* < 0.001).

The differences in craniofacial morphology between the OSA group and the control group adjusted for age, gender, and BMI are shown in Table 1. The OSA patients had a significantly more retrognathic maxilla (*p* = 0.02) and mandible (*p* = 0.032 and *p* = 0.009), a significantly larger beta-angle (*p* = 0.006), and a significantly smaller jaw angle (*p* = 0.045) than the control group.

The differences in posterior cranial fossa dimensions, skull thickness, and nasal bone length between the OSA group and the control group adjusted for age, gender, and BMI are shown in Table 2. The length of the posterior cranial fossa was significantly larger in all linear measurements in the OSA group (*p* = 0.003, *p* = 0.001, *p* = 0.044) and the depth was significantly increased (*p* < 0.001) compared to the control group. No significant differences in skull thickness and nasal bone length were found between the groups. Furthermore, no significant differences in the occurrence of morphological deviations in the sella turcica were found between the groups (OSA patients: 12 (23.5%), controls: 25 (33.8%), *p* = 0.235).

The differences in head posture between the OSA group and the control group adjusted for age, gender, and BMI are shown in Table 3. The OSA patients had a significantly more extended head posture (NSL/CVT, *p* < 0.001; NL/CVT, *p* < 0.001; NSL/OPT, *p* < 0.001; NL/OPT, *p* < 0.001) and a more forward inclination of the upper spine (OPT/HOR, *p* < 0.001; CVT/HOR, *p* = 0.001).

The differences in upper spine morphological deviations between the OSA group and the control group adjusted for age, gender, and BMI are shown in Table 4. The total fusion anomalies (fusion and block fusion) occurred significantly more often in the OSA group compared to the controls (*p* = 0.05).

## 4. Discussion

The present study aimed to compare craniofacial morphology, including skull thickness, posterior cranial fossa dimensions, nasal bone length, morphological deviations in sella turcica, head posture, and morphological deviations in the upper spine between OSA patients and healthy controls with neutral occlusion. The skull thickness, sella turcica morphology, and nasal bone length have not previously been investigated in OSA patients compared to healthy controls. The present study may therefore contribute to finding new phenotypic characteristics in OSA patients, which may be valuable in the diagnostics and treatment of OSA patients.

In the present study, the OSA patients were all diagnosed with an AHI > 5 using polygraphy and the gender ratio was 2/3 male and 1/3 female. The gender ratio is highly representative of the occurrence of OSA in society as studies have shown that the occurrence of OSA is greater in men than in women, with a ratio of 2:1 [6,15]. Furthermore, the BMI in the OSA patient group was significantly increased compared to the controls who had a BMI within normal values [34]. An increased BMI and a predominantly male group are often seen in OSA patients [1,6]. Thus, the OSA group in the present study is considered representative of the adult OSA population in general. The gender distribution in the control group was with a higher number of women than men, with a lower age and BMI within normal values than in the OSA patients. The differences in age, gender, and BMI between the groups were adjusted statistically to minimize the bias of the differences between the groups. The control group was not determined through polygraphy, whereby some subjects in the control group may have suffered from a non-symptomatic form of OSA [35]. However, a careful anamnesis was conducted, which did not show any signs of sleep-related breathing disorders in the controls [1,6]. The sample size of the two groups in the present study was considered sufficient according to the power calculation.

The methods used in the present study to measure the craniofacial morphology and upper spine were all standard methods with low method error and high reliability in accordance with previous studies [24,26,27,28,29,36,37,38,39]. In the present study, the lateral cephalograms were taken in natural head posture using the mirror position, which is a standardised and reproducible method [24,25]. Previous studies have used the Frankfurt Horizontal plane to position the subjects in the cephalostat, but then the individual head posture variation disappears [40] and cannot be useful in studies analysing head posture [18].

The differences in the retrognathia of the maxilla and mandible in OSA patients were in accordance with previous studies [15,18,41]. The retrognathia of the jaws is presumed to be associated with a reduction in the anterior–posterior dimensions of the airways [21,41] and may thereby contribute to the collapse of upper airways observed in OSA patients [6]. However, some studies have not found a difference in the prognathism of the jaws compared to controls [19,21,42]. Disagreements between the results of previous studies and the present study is probably caused by the multifactorial aetiology behind OSA [15] and that obstruction of upper airways may to a greater degree be caused by the dimensions and volume of the soft tissue in combination with the placement of the jaws [8,43].

In the present study, the posterior cranial fossa dimensions were significantly different between the OSA patients and controls. The length and the depth were significantly increased in the OSA patients compared to the controls. No previous studies have compared the posterior cranial fossa dimensions in OSA patients with a healthy control group. A previous study found an association between the posterior cranial fossa dimensions and morphological deviations of the upper spine [28] and morphological deviations of the upper spine is often found in OSA patients [14] in accordance with the present study.

In the present study, OSA patients had a more extended head posture compared to controls, which was in agreement with previous studies [16,44,45]. The extended head posture found in OSA patients may possibly be caused by the aetiology behind OSA in terms of the obstruction of upper airways and the increased air resistance. It has been found that extension of the head can reduce the upper airway resistance and the extension of the head may therefore by a physiological compensation mechanism in OSA patients [17,46].

The occurrence of morphological deviations of the upper spine was small in the present study. Still, the morphological deviations in the upper spine occurred significantly more often in OSA patients compared to controls, which was in accordance with previous studies [14]. Furthermore, an association between morphological deviations in the upper spine and a more extended head posture has been shown [26], which is in accordance with the present study. Moreover, it has previously been found that OSA patients with morphological deviations in the upper spine had retrognathia of the jaws and extended head posture compared to OSA patients without upper spine morphological deviations [15]. The similar craniofacial morphology was found in the OSA group of the present study. A study has also shown that OSA patients with morphological deviations in the upper spine respond poorly to MAD treatment [22]. This indicates that the aetiology behind OSA in patients with deviations in the upper spine can deviate from OSA patients without deviations in the upper spine [15].

In the present study, no significant differences in skull thickness, nasal bone length, or sella turcica morphology were found between the OSA patients and the controls. These structures in OSA patients have not previously been reported in the literature. The nasal bone length, the anterior part of the sella turcica, and skull thickness share the same embryonic origin associated with the frontonasal development field [47,48]. The significant differences in posterior cranial fossa dimensions, head posture, and upper spine morphological deviations found in the present study all belong to the cerebellar-cervical development field [47,48]. Therefore, the reason for not having found significant differences in the OSA group concerning skull thickness, length of the nasal bone, or deviations in sella turcica morphology could be related to the difference in embryonic origin.

## 5. Conclusions

The present study found significant deviations in the OSA patients as regards craniofacial morphology with the retrognathia of the maxilla and the mandible, larger beta-angle, significantly smaller jaw angle, and increased length and depth in the posterior cranial fossa compared to the control group.

Furthermore, the OSA patients had a significantly more extended and forward-inclined head posture and morphological deviations in the upper spine occurred significantly more often in OSA patients compared to controls. There were no significant differences in skull thickness, nasal bone length, and morphological deviations in sella turcica between the two groups. The results of the present study may prove valuable in the diagnostics of OSA patients and in considerations regarding etiology and phenotypic differentiation of OSA patients.

## Figures and Tables

**Table 1:** Description and comparison of the craniofacial morphology in the two groups adjusted for age, gender, and BMI.

Variable (Degrees)	OSA					Control					Comparison
	N	Min	Max	Mean	SD	N	Min	Max	Mean	SD	*p* Value
Cranial variables											
n-s-ba	51	118.6	138.3	130.85	4.87	74	120.4	141	131.68	4.46	0.512
n-s-ar	51	109.7	131.2	121.8	4.98	74	111.3	134.8	122.37	5.1	0.413
Sagittal variables											
ML/RL	51	103.8	132.2	117.76	6.01	74	105.4	134.2	118.16	6.31	0.045 *^cd^
MBL/ML	51	15.7	32.3	22.5	3.22	74	15.7	28.4	21.09	2.42	0.006 **^b^
s-n-ss	51	76.1	92.7	82.58	3.72	74	75.8	92.6	83.76	3.57	0.020 *
s-n-pg	51	68.9	89.5	80.67	4.44	74	75	93.7	82.36	3.89	0.032 *^a^
s-n-sm	51	69.1	86.2	79.24	4.10	74	74.2	91.5	81.12	3.86	0.009 *^a^
ss-n-pg	51	−7.8	10.5	1.75	3.31	74	−6.1	7.7	1.23	2.98	0.761
ss-n-sm	51	−3.2	9.5	3.35	2.52	74	−4	9.1	2.62	2.41	0.450
Vertical variables											
NSL/NL	51	−0.4	12.9	5.61	3.19	74	−4.6	13.2	5.95	4.09	0.792
NSL/ML	51	11	42	27.35	6.99	74	15	41.7	27.95	5.61	0.677
NL/ML	51	7.6	33.3	21.74	5.29	74	11.9	33.3	21.89	5.06	0.496
NSL/OLf	51	−5.2	26.6	12.93	6.3	74	−0.4	73.7	13.9	8.8	0.426

N: number, Min: minimum, Max: maximum, Mean: mean value, SD: standard deviation, * (*p* < 0.05), ** (*p* < 0.01), ^a^ gender effect (*p* ≤ 0.01), ^b^ age effect (*p* ≤ 0.05), ^c^ age effect (*p* < 0.01), and ^d^ BMI effect (*p* ≤ 0.01).

**Table 2:** Description and comparison of skull thickness, nasal bone length, and posterior cranial fossa dimensions in the two groups adjusted for age, gender, and BMI.

Variable	OSA					Control					Comparison
	N	Min	Max	Mean	SD	N	Min	Max	Mean	SD	*p* Value
Skull thickness (mm)											
Frontal	50	3.5	11.4	6.73	1.57	72	4.0	11.2	6.61	1.45	0.733
Parietal	48	4.4	11.5	7.83	1.55	71	4.8	11.9	7.35	1.54	0.839
Occipital	49	2.9	14.4	7.3	2.59	74	3.4	11.8	6.78	1.99	0.991
Nasal bone length (mm)											
Nasal bone length	50	12.2	31.1	21.4	4.25	74	16.5	27.9	21.68	3.27	0.794
Posterior cranial fossa dimensions											
Iodp-iop (mm)	51	26.9	50.4	37.75	5.34	74	27.7	49.3	34.66	3.95	0.044 *^c^
Iodp-iod (mm)	51	19.9	41.0	33.7	3.73	74	25.2	36.4	31.02	2.55	˂0.001 ***
Iop-s-iod (Degrees)	51	22.4	37.6	29.1	3.70	74	23.3	34.1	28.29	2.59	0.062
s-iod (mm)	51	58.7	80.2	70.13	4.72	74	58.7	76.3	65.66	3.65	0.001 ***^a^
s-iop (mm)	51	85.2	118.9	98.99	7.31	74	82.3	106.4	92.47	4.91	0.003 **^ab^

N: number, Min: minimum, Max: maximum, Mean: mean value, SD: standard deviation, * *p* < 0.05, ** *p* < 0.01, *** *p* < 0.001, ^a^ gender effect (*p* < 0.001), ^b^ BMI effect (*p* < 0.01), ^c^ BMI effect (*p* < 0.001).

**Table 3:** Description and comparison of head posture in the two groups adjusted for age, gender, and BMI.

Variable (Degrees)	Control					OSA					Comparison
	N	Min	Max	Mean	SD	N	Min	Max	Mean	SD	*p* Value
Cranio-vertical											
NSL/VER	74	85.10	109.1	98.2	4.71	51	87.0	115.4	99.16	6.09	0.391
NL/VER	74	83.20	102.2	92.23	4.62	51	83.2	105.7	93.54	5.37	0.468
Cranio-cervical											
NSL/OPT	74	78.40	111	94.40	6.9	51	85.8	114.9	100.86	8.26	˂0.001 ***^b^
NSL/CVT	64	85.2	114.1	100.72	6.23	47	92	121	106.79	8.05	˂0.001 ***^ab^
NL/OPT	74	75.8	106.1	88.53	6.73	51	81.7	110	95.24	7.36	˂0.001 ***^b^
NL/CVT	64	83.3	107.2	94.44	6.02	47	87.3	116	100.90	7.23	˂0.001 ***^b^
Cervico-horisontal											
OPT/HOR	74	75.5	107.3	94.04	6.5	51	74.8	98.3	88.33	6.05	˂0.001 ***^b^
CVT/HOR	64	74.4	102.7	87.63	6.08	47	71.4	94.4	82.57	5.56	0.001 ***
Cervical curvature											
CVT/OPT	64	−0.5	12.8	6.37	2.65	47	−2.4	12.5	5.4	2.73	0.498

N: number, Min: minimum, Max: maximum, Mean: mean value, SD: standard deviation, *** *p* < 0.001, ^a^ gender effect (*p* < 0.05), and ^b^ BMI effect (*p* < 0.05).

**Table 4:** Description and comparison of morphological deviations in the upper spine in the two groups adjusted for age, gender, and BMI.

	OSA		Control		Comparison
	N	%	N	%	*p* Value
Fusion anomalies					
Fusion (C2-C3 or C4-C5)	16	31.4%	12	16.2%	0.117
Block fusion	5	9.8%	0	0	0.465
Total number of fusion anomalies	21	41.2%	12	16.2%	0.05 **^ab^
Posterior arch deficiency (PAD)					
Partial cleft	8	15.7%	10	1f3.5%	0.614
Dehiscence	2	3.9%	0	0	0.991
Total number of PADs	10	19.6%	10	13.5%	0.758
More than 1 deviation	6	11.8%	2	2.7%	0.341
Total upper spine deviations	25	49%	20	27%	0.271

N: number, %: percentage of the group, ** *p* < 0.05, ^a^ gender effect (*p* < 0.001), and ^b^ age effect (*p* < 0.05).

## Data Availability

The data presented in this study are available on request from the corresponding author. The data are not publicly available due to privacy.
